# Colistin-resistant *Escherichia coli* with *mcr* genes in the livestock of rural small-scale farms in Ecuador

**DOI:** 10.1186/s13104-019-4144-0

**Published:** 2019-03-04

**Authors:** Yoshimasa Yamamoto, Manuel Calvopina, Ricardo Izurieta, Irina Villacres, Ryuji Kawahara, Masahiro Sasaki, Mayumi Yamamoto

**Affiliations:** 1 Graduate School of Pharmaceutical Sciences, Osaka University, 1-6 Yamadaoka, Suita, Osaka 565-0871 Japan; 2 Universidad De Las Americas, Quito, Ecuador; 3 College of Public Health, University of South Florida, Tampa, FL USA; 4 Osaka Institute of Public Health, Osaka, Japan; 5 Health Administration Center, Gifu University, Gifu, Japan

**Keywords:** Colistin-resistant bacteria, *mcr*, Livestock, *Escherichia coli*, Ecuador

## Abstract

**Objective:**

Emergence and dissemination of colistin-resistant (Co-R) bacteria harboring mobile colistin resistance genes pose a threat for treatment of infections caused by multi-drug resistant bacteria. Although the worldwide spread of Co-R bacteria is known, the precise state of Co-R bacterial dissemination in livestock of Andean countries remains unclear. Therefore, we investigated *mcr*-containing Co-R *Escherichia coli* dissemination in livestock on small-scale farms in two socioecologically different regions of Ecuador: the Amazonian rain-forest and the Pacific Coast.

**Results:**

Sixty-six rectal swab samples from 34 pigs and 32 chickens, from five farms in the two regions, were assessed for the dissemination of Co-R *E. coli* using the selective medium CHROMagar™ COL-APSE. *mcr*-containing Co-R *E. coli* were detected in the specimens at a high rate (47%; 31/66), but the detection rates of the two regions were not statistically different. Both chickens and pigs showed similar detection rates. All Co-R *E. coli* isolates harbored *mcr*-*1*. The minimum inhibitory concentrations of colistin were ≥ 8 mg/L, and 67.7% (21/31) of the Co-R isolates were multi-drug resistant. Pulsed-field gel electrophoresis revealed the limited relation between isolates. Thus, we revealed the high rate of widespread dissemination of Co-R bacteria in livestock regardless of the socioecological conditions in Ecuador.

## Introduction

Antibiotic abuse in livestock is considered to be related to the emergence and dissemination of antimicrobial-resistant (AMR) bacteria [1]. In fact, many AMR bacteria have been reported to spread in livestock and their products in regions where many antimicrobial agents are used extensively [2]. Furthermore, recent studies have shown that bacteria resistant to colistin, which is the last-resort antibiotic for treatment of intractable infections with multi-drug resistant (MDR) bacteria, have emerged and are spreading in these regions [3]. In particular, the emergence of colistin-resistant (Co-R) bacteria with mobile colistin resistance genes, *mcr*, has raised a serious concern for the treatment of infectious diseases because of the transmissibility of these genes between bacterial strains and species. However, recent studies have shown that the prevalence of Co-R bacteria with *mcr* in livestock varies with the region and country [4–7] probably because of the different antibiotic usage conditions in different regions.

Limited studies have focused on the prevalence of Co-R bacteria in livestock of Ecuador [8], which is characterized by a unique and heterogeneous ecology compared to that in the European and Asian countries. In addition, the prevalence of Co-R bacteria with *mcr* in these regions is not well known. Therefore, we investigated the spread of *mcr*-containing Co-R bacteria in livestock of Ecuador by selecting two representative rural regions: the Amazonian rain-forest and the Pacific Coast.

## Main text

### Materials and methods

#### Sample collection

We obtained 66 rectal stool swab samples from livestock, including those from pig (n = 34) and chicken (n = 32) in December 2017. Of these, 35 (16 chicken, 19 pig) were obtained from three farms in the Pacific Coast Santo Domingo and 31 (16 chicken, 15 pig) were obtained from two farms in the Amazonian rain-forest Puyo, Ecuador. The stool swab samples in Cary-Blair medium (Eiken Chemical, Tokyo, Japan) were stored in a cooler box until the isolation of bacteria on a culture plate for up to 24 h.

#### Bacterial isolation and susceptibility testing

The stool samples were directly inoculated on a selective agar medium CHROMagar™ COL-APSE (CHROMagar, Paris, France) for isolation of Co-R Gram-negative bacteria. After 24 h of incubation at 37 °C, one representative *Escherichia coli*-like colony was isolated and characterized further for bacterial identification using biochemical tests with triple sugar iron slants, motility-indole-lysine medium (BD, New Jersey, USA), cellobiose lactose indole β-glucuronidase medium (Nissui, Tokyo, Japan), and the API 20E system (bioMerieux, Marcy-l’Étoile, France).

Colistin susceptibility of the isolates was evaluated by ETEST^®^ (bioMerieux), according to the manufacturer’s protocol. The susceptibility to other antibiotics, including ampicillin, cefoxitin, cefotaxime, ceftazidime, meropenem, streptomycin, kanamycin, gentamicin, ciprofloxacin, nalidixic acid, tetracycline, chloramphenicol, fosfomycin, and sulfamethoxazole-trimethoprim, was tested using the disc-diffusion method following the standard procedure of the Clinical and Laboratory Standards Institute (Wayne, PA, USA), as described previously [7].

#### Detection of *mcr* genes

Bacterial DNA was extracted by boiling the bacterial suspension in tris(hydroxymethyl)aminoethane-EDTA buffer. The presence of the colistin resistance genes, *mcr*-*1,* -*2,* -*3,* -*4*, and -*5*, was detected by PCR using bacterial DNA and sequencing of the resulting products, as described previously [9].

#### Clonality study

Pulsed-field gel electrophoresis (PFGE) of *Xba*I-digested genomic DNA samples from the isolates was performed using a CHEF-DR II System (Bio-Rad, Hercules, CA, USA) following the PulseNet protocol [10].

#### Statistical analysis

Statistical analysis was performed using the Student’s *t* test. The significance level was set at *P *< 0.05.

### Results and discussion

Of the 35 samples (16 chicken, 19 pig) obtained from the three farms in Santo Domingo, 18 samples (51.4%; 9 chicken, 9 pig) showed the presence of Co-R bacteria when inoculated on the selective medium for Co-R bacteria (Table 1A). One representative *E. coli*-like colony was isolated and identified by a standard biochemical method. Of the 31 samples (16 chicken, 15 pig) obtained from the two farms in Puyo, Co-R *E. coli* were found in 13 samples (41.9%; 6 chicken, 7 pig). Although, there is a 10.5% difference in the Co-R *E. coli* detection rates between the two regions, this difference was not statistically significant. Moreover, the detection rates of Co-R *E. coli* in chickens and pigs, were similar (Table 1B).

**Table 1:** Detection of colistin-resistant *Escherichia coli* in stool samples of livestock

(A) Detection of colistin-resistance in farms												
		City	Santo Domingo						Puyo			
		Farm^a^	A		B		C		D		E	
	Total	Livestock	Pig	Chicken	Pig	Chicken	Pig	Chicken	Pig	Chicken	Pig	Chicken
No. of samples tested	66		0	12	5	4	14	0	0	16	15	0
No. of colistin-resistant *E. coli* positive samples	31		0	6	0	3	9	0	0	6	7	0
No. of samples positive/No. of samples tested	31/66 (47.0%)		18/35 (51.4%)						13/31 (41.9%)			

(B) Detection of colistin-resistance in livestock												
	Pig	Chicken										
No. of samples tested	34	32										
No. of colistin-resistant *E. coli* positive samples	16 (47.1%)	15 (46.9%)										

^a^A–E means individual farm

A previous report on the prevalence of Co-R bacteria in Ecuador states that 16% (n = 10) of *Salmonella* isolates from poultry slaughterhouses were Co-R [11]. This rate is different than the results obtained in this study probably because of difference in the sampling method used and/or difference in the target bacteria. Although the number of specimens in the present study was limited, it revealed a high detection rate (47%) of Co-R bacteria in livestock, regardless of the differences in the socioecological conditions in the two representative regions of Ecuador, the Amazonian rain-forest and the Pacific Coast. To the best of our knowledge, this is the first report of such a high rate of Co-R bacteria in Ecuador, a country located in the Andean region. The reason of such high dissemination of Co-R bacteria in the livestock of Ecuador is not clear; however, it seems to be caused by the use of colistin formula feed during breeding, similar to the practice followed in Asian countries [12]. Indeed, we found that the commercial chicken and pig feeds available in the markets of the two regions studied contained colistin. This leads to colistin pressure on the microbiota of the livestock in farms irrespective of the socioecological development level or the size and geographical location of the farm.

The analysis of genes *mcr*-*1* to -*5* by PCR in Co-R *E. coli* isolates revealed that all Co-R *E. coli* harbored only *mcr*-*1* and the minimum inhibitory concentration of colistin was ≥ 8 mg/L. Currently, 8 *mcr* genes, *mcr*-*1* to *mcr*-*8*, have been identified [13]. The results of present study are consistent with the previous findings that *mcr*-*1* is ubiquitously present in various Enterobacteria of different origins in many countries [13, 14].

The test for susceptibility of Co-R isolates to other antibiotics showed that 67.7% of the 31 isolates were MDR bacteria, defined as resistance to at least one antibiotic drug in three or more antibiotic classes [15]. The isolates were resistant to an average 3.0 (range, 1 to 6) antibiotics. No isolate was resistant to carbapenem and none was identified as an extended-spectrum β-lactamase-producing isolate. This abundance of MDR bacteria in livestock is similar to that observed in the livestock products in Asian countries [16]. Such abundance observed in previous studies and the present study may be attributed to similar livestock breeding conditions with respect to the use of antibiotics in rural small-scale farms regardless of the geographical difference.

**Fig. 1:**
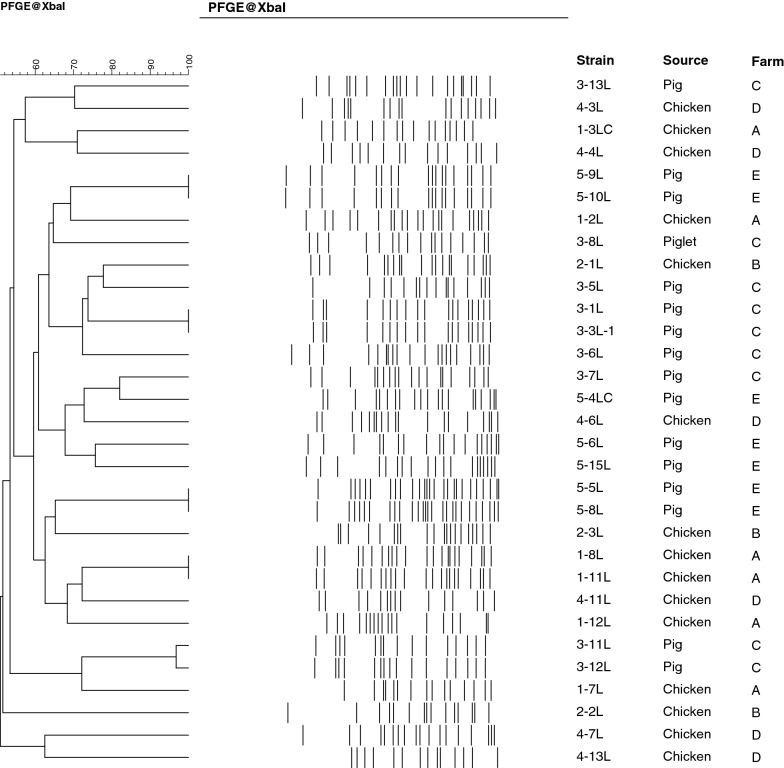
Dendrogram of pulsed-field gel electrophoresis patterns among colistin-resistant *Escherichia coli* isolates

PFGE analysis of Co-R *E. coli* isolates showed that similar clonal strains were isolated from the same livestock species on the same farm in five cases; however, the lineage of most isolates among farms and livestock was diverse (Fig. 1). These results suggest the limitation of clonal expansion in these highly prevalent Co-R *E. coli* in the livestock on the farms investigated. In contrast, the diversity of Co-R *E. coli* isolates in the livestock suggests the horizontal transmission of *mcr*-*1* among the intestinal microbiota of the livestock and/or environmental microbes on these farms.

Widespread dissemination of Co-R bacteria in livestock can be a high risk in communities, including human residents; therefore, the results obtained in this study warrant a full-scale surveillance operation and public health efforts to control the spread of Co-R bacteria.

## Limitations

The main limitation of this study is that the number of samples was small to justify the complete analysis of the prevalence of Co-R bacteria in the livestock of the two regions. Nevertheless, the observation of a high detection rate of Co-R bacteria with *mcr*-*1* in the livestock in small farms in Ecuador is enough to highlight the threat to local communities and to raise a public health concern. Further analysis on the spread of Co-R bacteria in different communities such as human residents and environments, as well as the spread of *mcr* genes in infection-causing bacteria should be monitored.

## Abbreviations

AMR: antimicrobial-resistant
Co-R: colistin-resistant
MDR: multi-drug resistant
PFGE: pulsed-field gel electrophoresis
